# Distinct clinical features of transplanted children with Parvovirus B19 infection

**DOI:** 10.1186/s12985-024-02380-4

**Published:** 2024-05-10

**Authors:** Ran Jia, Lingfeng Cao, Lijuan Lu, Huaqing Zhong, Menghua Xu, Pengcheng Liu, Xunhua Zhu, Liyun Su, Jin Xu

**Affiliations:** 1 Department of Clinical Laboratory, Children’s Hospital of Fudan University & National Children’s Medical Center, Shanghai, 201102 China; 2 Department of Pediatric Institute, Children’s Hospital of Fudan University & National Children’s Medical Center, Shanghai, China; 3 Shanghai Institute of Infectious Disease and Biosecurity, Fudan University, Shanghai, China

**Keywords:** Parvovirus B19, Transplantation, Children, Clinical features

## Abstract

**Background:**

The immature and suppressed immune response makes transplanted children a special susceptible group to Parvovirus B19 (PVB19). However, the clinical features of transplanted children with PVB19 infection haven’t been comprehensively described.

**Methods:**

We searched the medical records of all the transplant recipients who attended the Children’s Hospital of Fudan University from 1 Oct 2020 to 31 May 2023, and reviewed the medical literature for PVB19 infection cases among transplanted children.

**Results:**

A total of 10 cases of PVB19 infection were identified in 201 transplanted children at our hospital, and the medical records of each of these cases were shown. Also, we retrieved 40 cases of PVB19 infection among transplanted children from the literature, thus summarizing a total of 50 unique cases of PVB19 infection. The median time to the first positive PVB19 DNA detection was 14 weeks post-transplantation. PVB19 IgM and IgG were detected in merely 26% and 24% of the children, respectively. The incidence of graft loss/dysfunction was as high as 36%. Hematopoietic stem cell transplant (HSCT) recipients showed higher PVB19 load, lower HGB level, greater platelet damage, lower PVB19 IgM/IgG positive rates, and more graft dysfunction than solid-organ transplant (SOT) recipients, indicating a more incompetent immune system.

**Conclusions:**

Compared with the published data of transplanted adults, transplanted children displayed distinct clinical features upon PVB19 infection, including lower PVB19 IgM/IgG positive rates, more graft dysfunction, and broader damage on hematopoietic cell lines, which was even more prominent in HSCT recipients, thus should be of greater concern.

## Background

Parvovirus B19 (PVB19) is a single-stranded DNA virus that infects the majority of humans [1]. Serologic studies revealed that 60–90% of adults have antibodies against PVB19 [2]. PVB19 belongs to the Erythrovirus genus, the name of which describes its unique erythroid progenitor cell tropism [2]. The transmission routes for PVB19 are various, including respiratory secretions, donor grafts, transfusion of blood products, and maternal-neonatal transmission [2, 3]. In healthy subjects, PVB19 infection is a commonly asymptomatic or acute self-limited disease manifested as mild anemia, flu-like symptoms, infectious erythema, or arthropathy. But in immunocompromised patients, especially the transplant recipients, PVB19 infection could lead to refractory anemia, pancytopenia, pure red cell aplasia, transient aplastic crisis, and rarely hemophagocytic lymphohistiocytosis [4, 5], which can be severely detrimental to patients’ health. However, PVB19-infected transplanted children usually lack typical symptoms [6]. What’s worse, rare studies focused on the pediatric group, and the spectrum of clinical manifestations of PVB19-infected transplanted children has not been well characterized yet.

Here in this study, we summarized the clinical information of PVB19-infected transplanted children at our hospital and in the literature, totaling 50 cases. Afterwards, we compared the clinical profile of the pediatric recipients with those of adult recipients, and identified distinct clinical characteristics of them, thus providing references to the diagnosis and assessment of PVB19 infection among transplanted children.

## Methods

### Patients

To characterize the epidemiology and clinical spectrum of post-transplant PVB19 infection, we reviewed the medical records of all the pediatric transplant recipients visiting the Children’s Hospital of Fudan University from 1 Oct 2020 to 31 May 2023. PVB19 DNA and antibody detection was ordered by clinicians as routine surveillance after transplantation. Although patients were asked to schedule follow-up appointments at least once a month after transplantation, they may do so earlier or later due to illness (e.g., infection) or other reasons (e.g., transportation and distance). PVB19 infection was defined as the positive detection of PVB19 DNA in patients’ plasma (EDTA-anticoagulated whole blood) during the follow-up. Once a patient is determined to be PVB19 DNA positive, the clinician will schedule PVB19 DNA monitoring at one to two-week intervals during the hospitalization until the patient recovers, or his/her PVB19 DNA turns negative, or other uncontrollable causes (e.g., the patient’s non-cooperation) arise. The study was reviewed and approved by the Ethics Committee of the Children’s Hospital of Fudan University on Aug 2022 (Approval Number: 2022(176)).

### PVB19 DNA and antibody detection

PVB19 DNA was tested in a clinical laboratory using real-time quantitative polymerase chain reaction (qPCR) (Sansure Biotech, China). The qPCR test was performed on one well per sample and the limit of detection was 4 × 10^2^ copies/ml. The standard curve was made based on five reference standards ranging from 4 × 10^3^ to 4 × 10^7^ copies/ml. To ensure the correction of the test results, quality control materials were set up for each batch of assay, including a positive control, a negative control, and a no-template control.

PVB19 IgM/IgG detection was performed using enzyme-linked immunosorbent assay (ELISA) (EUROIMMUN, Germany). Briefly, samples were added to microwells pre-coated with PVB19 antigen, followed by the addition of peroxidase-labeled anti-human IgM/IgG. The sample’s absorbance at 450 nm was collected using TMB as the substrate. The final result was determined by the ratio of the absorbance of the sample to that of the reference standard in the kit, with a cut-off value being 1.1 for both IgM and IgG.

### Review of the literature

A search of the English-language medical literature was performed using PubMed and Medline databases. Secondary references were reviewed. Twenty-five publications describing 42 unique cases of PVB19 infection among children after transplantation were retrieved [4, 6–29]. 2 cases from two published studies [12, 20] were removed from this study because PVB19 infection occurred before transplantation. The patient’s demographic information, type of transplant, onset of the disease, and clinical laboratory findings were collected.

### Statistical analysis

Counting data were presented as median with interquartile range (IQR), while categorical data were median with percentage. A chi-square test or Fisher’s exact test was used for the comparison of proportions for categorical variables. An unpaired Student’s *t*-test was used to test the differences in quantitative variables with a normal distribution. Otherwise, a Mann–Whitney *U* test was used. The statistical analyses were performed using GraphPad Prism software (version 6). Two-sided *p*-values of < 0.05 were considered significant.

## Results

### Case series of 10 patients

A total of 201 transplant cases were recorded in the Children’s Hospital of Fudan University from 1 Oct 2020, when our institution started the PVB19 DNA test, to 31 May 2023. PVB19 DNA was detected in the plasma of 10 out of 201 (5.0%) patients, with the positive rates of PVB19 DNA in kidney, liver, bone marrow, and lung transplant recipients being 5.0% (6/121), 3.6%(2/55), 8.7%(2/23) and 0%(0/2), respectively.

The demographical and clinical features of the 10 patients were shown in Table 1. All of the 10 children were male, with the median age at transplantation being 4y. The median time point of the first PVB19 DNA-positive detection after transplantation was 148 days, but half of the cases were found to be PVB19 DNA-positive in the first five weeks post-transplantation. The median of the highest plasma PVB19 DNA load was 9.37 × 10^7^ copies/ml. All the cases developed anemia, with a median minimum hemoglobin (HGB) level being 67 g/L. Leukopenia (60%) and thrombopenia (40%) were also common, with the medians of the lowest white blood cell (WBC) and platelet (PLT) counts being 3.3 × 10^9^/L and 163 × 10^9^/L, respectively. Pancytopenia was observed in 40% of cases. Graft loss or dysfunction was observed in half of the cases. Intravenous immunoglobulin (IVIG) was routinely used as a treatment modality for PVB19 infection.

**Table 1:** Demographic and clinical characteristics of 10 patients with PVB19 infection in transplanted children

Patient characteristics	Patient 1	Patient 2	Patient 3	Patient 4	Patient 5	Patient 6	Patient 7	Patient 8	Patient 9	Patient 10
Age at transplantation	15y	6y	8 m	4 m	9y	2y	7y	12y	1y	10 m
Sex	Male	Male	Male	Male	Male	Male	Male	Male	Male	Male
Type of transplant	Kidney	Kidney	HSCT	Liver	Kidney	Kidney	Kidney	Kidney	HSCT	Liver
First B19 DNA-positive detection, days post transplantation	261	31	324	7	2560	23	3020	21	445	34
Maximum viral load in the blood, copies/mL	3.14 × 10^4^	3.08 × 10^10^	7.15 × 10^8^	9.37 × 10^7^	2.56 × 10^3^	1.01 × 10^8^	5.28 × 10^4^	7.51 × 10^3^	5.39 × 10^10^	9.93 × 10^5^
Anemia	Yes	Yes	Yes	Yes	Yes	Yes	Yes	Yes	Yes	Yes
Lowest Hgb during B19 DNA positive, g/L	96	45	52	58	92	75	87	85	57	78
Leukopenia	Yes	Yes	Yes	Yes	No	No	No	Yes	Yes	No
Lowest WBC count, ×10^9^/L	2.4	1.3	2.83	1.8	5.3	4.13	4	3.72	2.94	4.7
Thrombopenia	No	Yes	Yes	Yes	No	No	No	No	Yes	No
Lowest PLT count, ×10^9^/L	184	73	14	67	251	292	189	168	62	157
Pancytopenia	No	Yes	Yes	Yes	No	No	No	No	Yes	No
Treatment	IVIG	IVIG	IVIG	IVIG	IVIG	IVIG	IVIG	IVIG	IVIG	IVIG
Graft dysfunction at disease onset	Yes	No	Yes	No	Yes	No	Yes	No	No	Yes

**Fig. 1:**
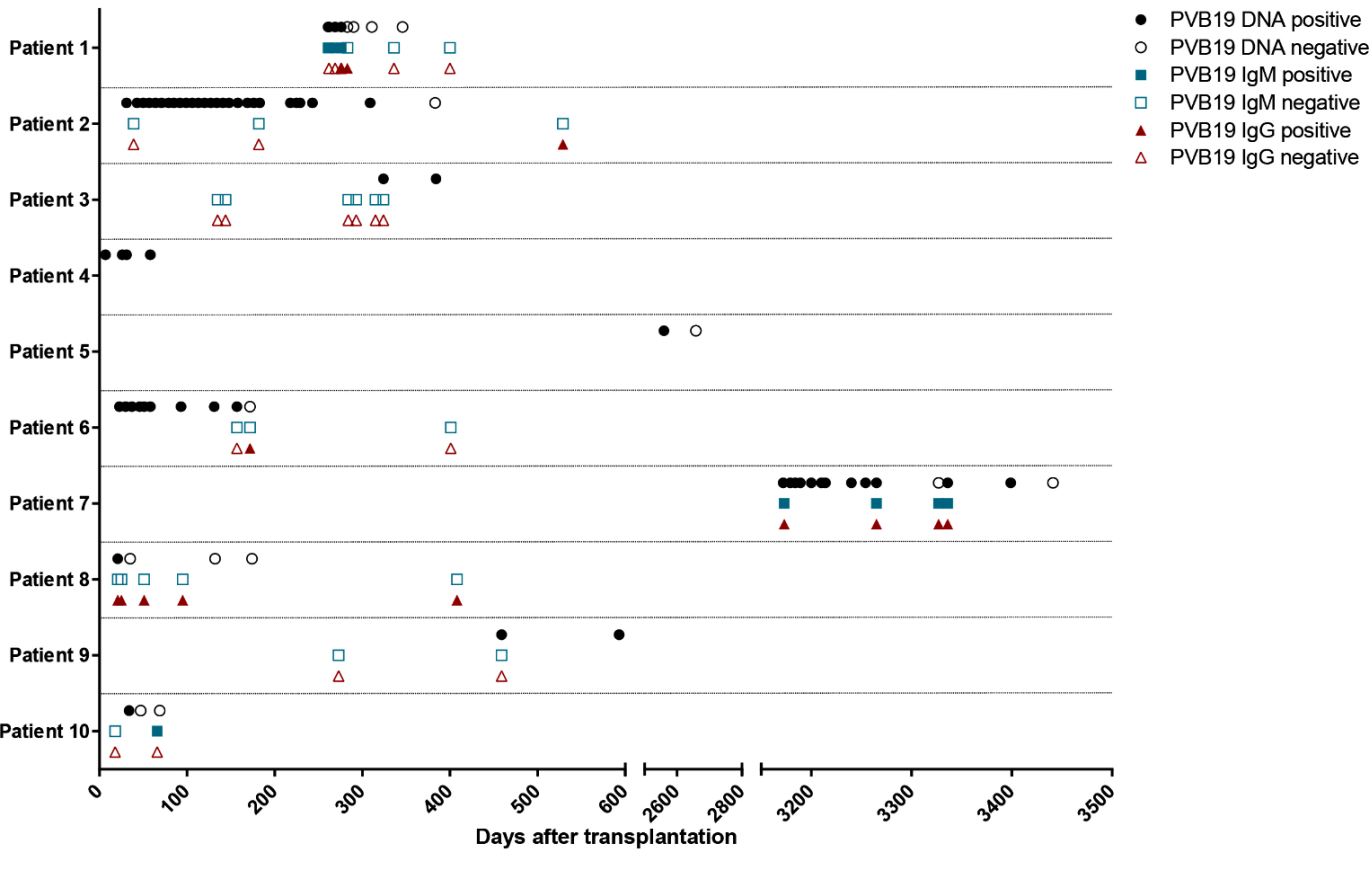
The timeline of the 10 patients’ PVB19 DNA/IgM/IgG test results. A total of 10 patients were finally found to be PVB19 DNA positive among 201 transplant cases in the Children’s Hospital of Fudan University from 1 Oct 2020 to 31 May 2023. The results of PVB19 DNA/IgM/IgG of the 10 cases were displayed during the course of the infection

**Fig. 2:**
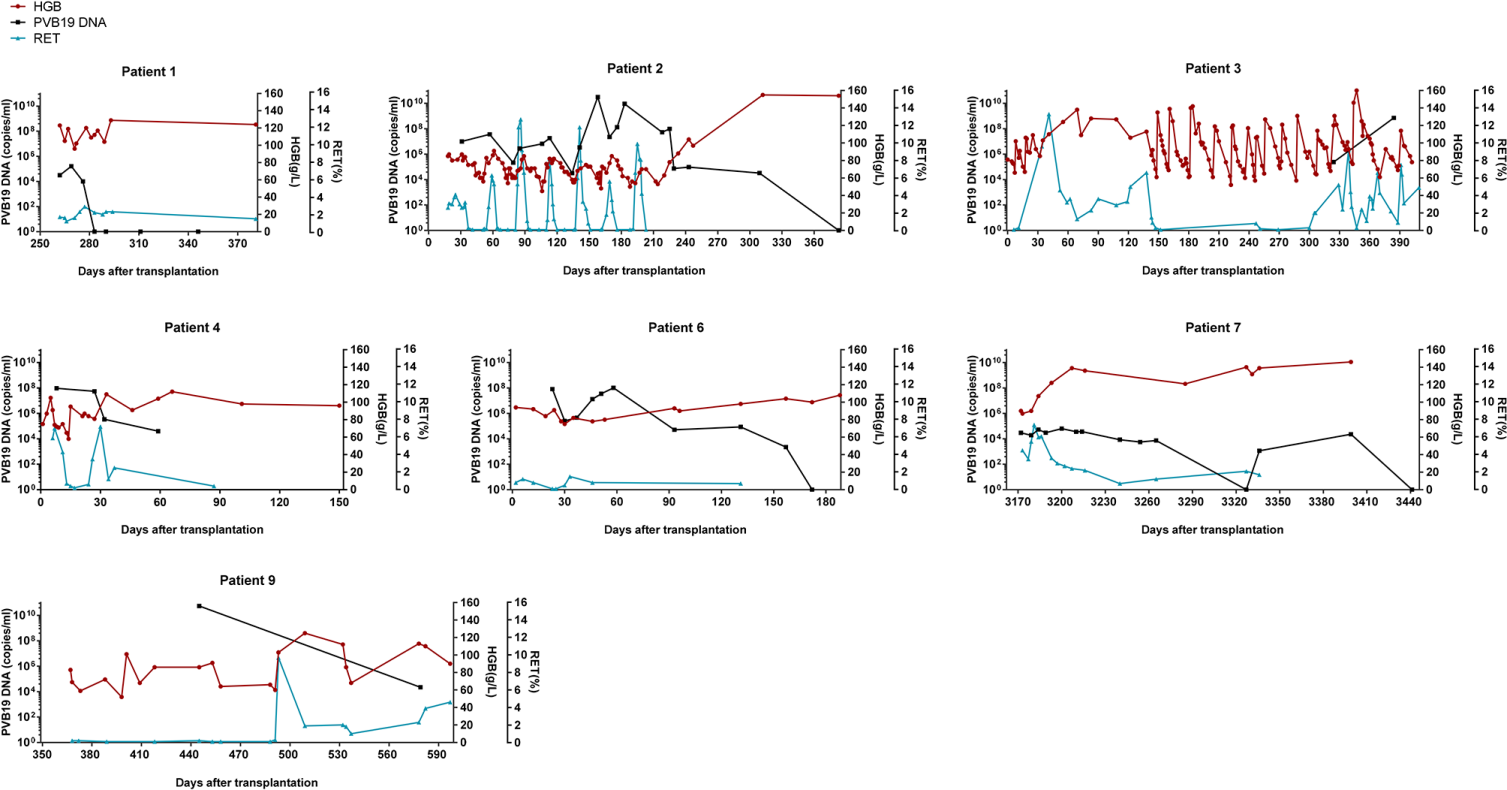
The dynamics of PVB19 DNA, HGB level, and RET percentage of seven transplanted children. Seven patients with more than one positive PVB19 DNA result were chosen to show the dynamics of PVB19 DNA, HGB level, and percentage of RET in red blood cells (RET%). HGB, hemoglobin. RET, reticulocyte

Next, we presented the timeline of the 10 patients’ PVB19 DNA/IgM/IgG tests and other virus detections (Fig. 1). Only 3 patients were positive for PVB19 IgM and 5 for IgG during the course of the disease. During the PVB19-positive period, a total of 4 patients were co-infected with other viruses including cytomegalovirus (CMV) (Patients 2 & 10), Epstein-Barr virus (EBV) (Patients 4 & 10), and BK virus (BKV) (Patients 2 & 8). Then we presented the dynamics of PVB19 DNA, HGB level, and reticulocyte percentage (RET%) of 7 patients with more than one positive PVB19 DNA result (Fig. 2), which showed persistent or recurrent anemia and a negative correlation between the viral load and HGB level or RET% of them.

### Review of the literature

To comprehensively capture the clinical features of PVB19 infection after transplantation in children, we reviewed the medical literature and finally enrolled 40 unique cases [4, 6–11, 13–19, 21–29]. Together with the 10 cases in our hospital, a total of 50 cases were summarized. 19 hematopoietic stem cell transplants (HSCT) and 31 solid organ transplant (SOT) were included in the population, with the latter including 14 kidney transplants, 13 liver transplants (including one liver and pancreas transplant), 3 heart transplants, and 1 lung transplant (Table 2). The majority of the children were boys (78.0%). The median time to the first PVB19 positive detection after transplantation was 14 weeks. PVB19 IgM and IgG were only detected in 13(26.0%) and 12(24.0%) cases, respectively. 36.0% of the cases developed graft dysfunction during PVB19 infection.

**Table 2:** Clinical characteristics of 50 children with PVB19 infection after SOT or HSCT

	All patients (*n* = 50)	SOT patients (*n* = 31)	HSCT patients (*n* = 19)	*P* value
Age of transplant, years	8(3 ∼ 11)	9(3 ∼ 11)	8(4 ∼ 13)	
No. of male/female	39/11	22/9	17/2	0.302
First B19 DNA-positive detection, weeks post transplantation	14(5 ∼ 47)	10(4 ∼ 136)	14(9 ∼ 28)	0.613
PVB19 IgM positive during the disease course	13(26.0%)	11(35.5%)	2(10.5%)	0.105
PVB19 IgG positive during the disease course	12(24.0%)	10(32.3%)	2(10.5%)	0.160
Maximum viral load in the blood, copies/ml	9.74 × 10^7^(4.75 × 10^4^∼1.19 × 10^10^)	5.28 × 10^4^(8.01 × 10^3^∼4.74 × 10^7^)	1.40 × 10^10^(7.26 × 10^9^∼4.21 × 10^10^)	0.001
Anemia	50(100.0%)	31(100.0%)	19(100.0%)	1.000
Lowest HGB during B19 DNA positive, g/L	68(54 ∼ 82)	78(58 ∼ 89)	58(54 ∼ 67)	0.012
Leukopenia	20(40.0%)	10(32.3%)	10(52.6%)	0.258
Lowest WBC count, ×10^9^/L	3.2(2.4 ∼ 4.9)	3.7(2 ∼ 4.9)	2.9(3 ∼ 3.6)	0.147
Thrombopenia	18(36.0%)	6(19.4%)	12(63.2%)	0.005
Lowest PLT count, ×10^9^/L	73(57 ∼ 168)	94(63 ∼ 176)	47(31 ∼ 58)	0.026
Pancytopenia	10(20.0%)	4(12.9%)	6(31.6%)	0.216
Graft dysfunction at disease onset	18(36.0%)	5(16.1%)	13(68.4%)	< 0.001

Next, we performed a comparison of HSCT and SOT recipients. The positive rate of PVB19 IgM was much lower in HSCT children (10.5%) than in SOT children (35.5%), which was also the case for PVB19 IgG (10.5% vs. 32.3%). Meanwhile, HSCT patients had a higher maximum viral load of PVB19 (1.40 × 10^10^ copies/ml) than SOT patients (5.28 × 10^4^ copies/ml). Anemia was observed in all the patients, but HSCT patients had much lower HGB levels (58(54 ∼ 67) g/L) than SOT patients (78(58 ∼ 89) g/L). Moreover, HSCT patients showed a higher incidence of thrombopenia (63.2%) and lower minimum PLT counts (47 × 10^9^/L) than SOT patients (19.4%, 94 × 10^9^/L). A total of 18 cases suffered with graft loss/dysfunction during PVB19 infection, with 5 (27.8%) being SOT recipients, and 13 (72.2%) being HSCT recipients. These data indicate that HSCT recipients developed more severe clinical manifestations than SOT recipients.

## Discussion

The immature and suppressed immune response makes transplanted children a special susceptible group to PVB19 infection. As reported, the rate of positive PVB19 infection is higher in pediatric transplant recipients than in their adult counterparts [30]. Moreover, the clinical manifestations of pediatric recipients lack specificity [30], highlighting the need for a comprehensive clinical profile of them. A study that included 98 PVB19-infected adult transplant recipients reported higher positive rates of PVB19 IgM (71.2%) and IgG (38%), but lower incidences of thrombopenia (21.2%), leukopenia (37.3%) and graft dysfunctions (10.4%) than the pediatric recipients in our study [5], indicating a less immunocompetent state and a greater damage on hematopoietic cell lines of the pediatric recipients.

Similar to children, the clinical manifestations of HSCT recipients in adults are also more severe than those of SOT recipients [5], for reasons that are unclear. Presumably, the primary diseases of HSCT patients include leukemia and immunodeficiency, etc., the immune systems of which are already vulnerable due to long-term chemotherapy or genetic defects [31]. Moreover, in order to allow for reconstruction of the hematopoietic and immune system, HSCT recipients are routinely pretreated with high-dose chemo- or radio-therapy to clear the malignant or abnormal cells in blood before transplantation, thus leading to a more severe bone marrow suppression and more fragile immune defense [31]. Hence, it is rational that HSCT patients displayed more severe clinical presentations. Besides therapeutic differences, the patients have primary PVB19 infection or not might also matter, since non-primary infections are supposed to show better clinical presentations due to the immune memory. Also, the discrepancies caused by different transmission routes and sampling times should not be ignored, as viral loads change dynamically based on the initial infection dose and the progression of the disease [1]. Collectively, further efforts are needed to figure out whether the above-mentioned factors contribute to the worse clinical manifestations of HSCT recipients.

PVB19 has a highly restrictive tropism of erythroid progenitor cells (EPCs) by binding to the neutral glycosphingolipid (GSL) globoside (Gb4) or P antigen, exhibiting cellular lysis and hemagglutinating activity [12, 32]. Seemingly paradoxically, PVB19 DNA has been detected in various organs of human [33, 34]. This phenomenon can be explained by the findings of Norja et al., who revealed that after the initial infection of PVB19, the viral genomes persist in solid tissues (e.g., skin, synovium, tonsil, or liver) for lifelong and provide a registry of one’s infectious encounters [35]. Another paradox was that the restricted tropism of PVB19 does not align with the wide expression of Gb4 in organs including heart, liver, kidney, ovary, and brain [15, 33]. Upon further exploration, Bieri et al. revealed that pH is an affinity switch that regulates the interaction of PVB19 and Gb4 [32], thus expressing Gb4 does not equal to the ability to infect PVB19. Also, Ning et al. identified the tyrosine protein kinase receptor UFO (AXL) as a co-receptor for PVB19-infected EPCs [36]. Also, human EPCs express high levels of AXL on the cell surface, while PVB19 nonpermissive cells express negligible amounts of AXL [36], echoing the highly restrictive tendency of the virus. Notably, AXL is also widely expressed in human pulmonary epithelial cells [37], resonating with the main respiratory transmission route of PVB19. Hence, further efforts are required to verify the role of Gb4 and AXL in PVB19 transmission and other mechanisms contributing to the narrow tropism of the virus.

There are also certain limitations of this study. First, although we retrospectively reviewed all the transplanted cases in our institution during a nearly three-years period, the number of PVB19-infected cases is still small. That’s why we subjected to the literature to improve the representativeness of our data. Second, we might have missed some cases or information in the literature, leading to possible bias. Last but not least, since this is a retrospective study, and the donor grafts and blood products weren’t routinely screened for PVB19 DNA in our country, thus we couldn’t determine whether PVB19 infection in our cases was via donor-to-recipient transmission.

## Conclusions

PVB19 infection is a rare but critical disease among transplanted patients. Our study suggests that PVB19-infected pediatric recipients suffered with more damaged hematopoietic cell lines and a more incompetent immune response than their adult counterparts, with HSCT recipients being more prominent, and thereby warrants greater concern. Also, our findings highlight the fact that DNA detection, rather than IgM/IgG tests, is more credible for the diagnosis of PVB19 infection in immunosuppressed transplanted children who may fail to mount virus-specific antibodies.

## Data Availability

Data sharing is not applicable to this article as no datasets were generated or analysed during the current study.

## Abbreviations

HSCT: Hematopoietic stem cell transplants
HGB: Hemoglobin
IVIG: Intravenous immunoglobulin
PLT: Platelet
PVB19: Parvovirus B19
RET: Reticulocyte
SOT: Solid organ transplant
WBC: White blood cell
